# Genome analysis of *Lachnoclostridium phocaeense* isolated from a patient after kidney transplantation in Marseille

**DOI:** 10.1016/j.nmni.2021.100863

**Published:** 2021-03-16

**Authors:** I. Dandachi, H. Anani, L. Hadjadj, S. Brahimi, J.-C. Lagier, Z. Daoud, J.-M. Rolain

**Affiliations:** 1) Aix Marseille Université, IRD, APHM, MEPHI, IHU-Méditerranée Infection, Marseille, France; 2) Aix Marseille Université, IRD, APHM, VITROME, IHU-Méditerranée Infection, Marseille, France; 3) Faculty of Medicine and Medical Sciences, Clinical Microbiology Laboratory, University of Balamand, Amioun, Beirut, Lebanon; 4) Clinical Microbiology and Infection Prevention, Michigan Health Clinics, Central Michigan University, Mount Pleasant, MI, USA

**Keywords:** Bacteria, culturomics, *Lachnoclostridium*, new species, taxonogenomics

## Abstract

*Lachnoclostridium phocaeense* is a new species in the genus *Lachnoclostridium*. *Lachnoclostridium phocaeense* is a Gram-positive anaerobic rod. This strain, Marseille-P3177^T^ (CSUR = P3177) with the below described genome was isolated from the urine sample of a women after kidney transplantation. The strain genome is 3 500 754 bp long with 50.62% G + C content and consists of a single contig (GenBank accession number NZ_LT635479.1).

## Introduction

*Lachnoclostridium* is a genus of Gram-positive, obligate anaerobic, spore-forming, motile bacteria. Organisms in this genus can grow in moderate ‘mesophilic’ as well as in extremely high ‘thermophilic’ temperatures, ranging from 20°C to 45°C and from 203°C to 633°C, respectively [1].

The *Lachnoclostridium* genus includes organisms from the *Lachnospiraceae* family and from several clostridial clusters such as *Clostridium* XIVa [1]. Clostridial cluster XIVa is known to make up a significant part of the human gut microflora [2]; it can exert anti-inflammatory effects and plays a role in homeostasis. In addition, via its components and metabolites, notably butyrate, clostridial cluster XIVa maintains intestinal health [3].

The human gut microbiota is a complex ecosystem that contains a variety of organisms including bacteria, fungi and viruses [4]. To explore this niche, bacterial cultures were used [5]; however, provided information on only the cultivable part of the humvan gut with a considerable fraction being uncultured. This is despite the advancement of molecular techniques such as metagenomics and 16S rRNA sequencing [6]. Recently, a new approach combining bacterial culturing under different conditions, matrix-assisted laser desorption/ionization time-of-flight mass spectrometry (MALDI-TOF-MS) and 16s rRNA sequencing, named culturomics, was implemented. Compared with metagenomics, this approach allows the cultivation of species corresponding to previously unassigned sequences [7].

Using a previously described taxonogenomic approach [8,9] combined with culturomics, we present here the phenotypic and genomic characteristics of a *Lachnoclostridium* novel species isolated from a patient admitted to the hospital in Marseille. This is part of the culturomics project, which aims to detect and isolate new bacterial species. The new species was deposited in the *Collection de Souches de l’Unite des Rickettsies* (CSUR, WDCM 875) under the number P3177 [10].

## Strain identification

**Fig. 1:**
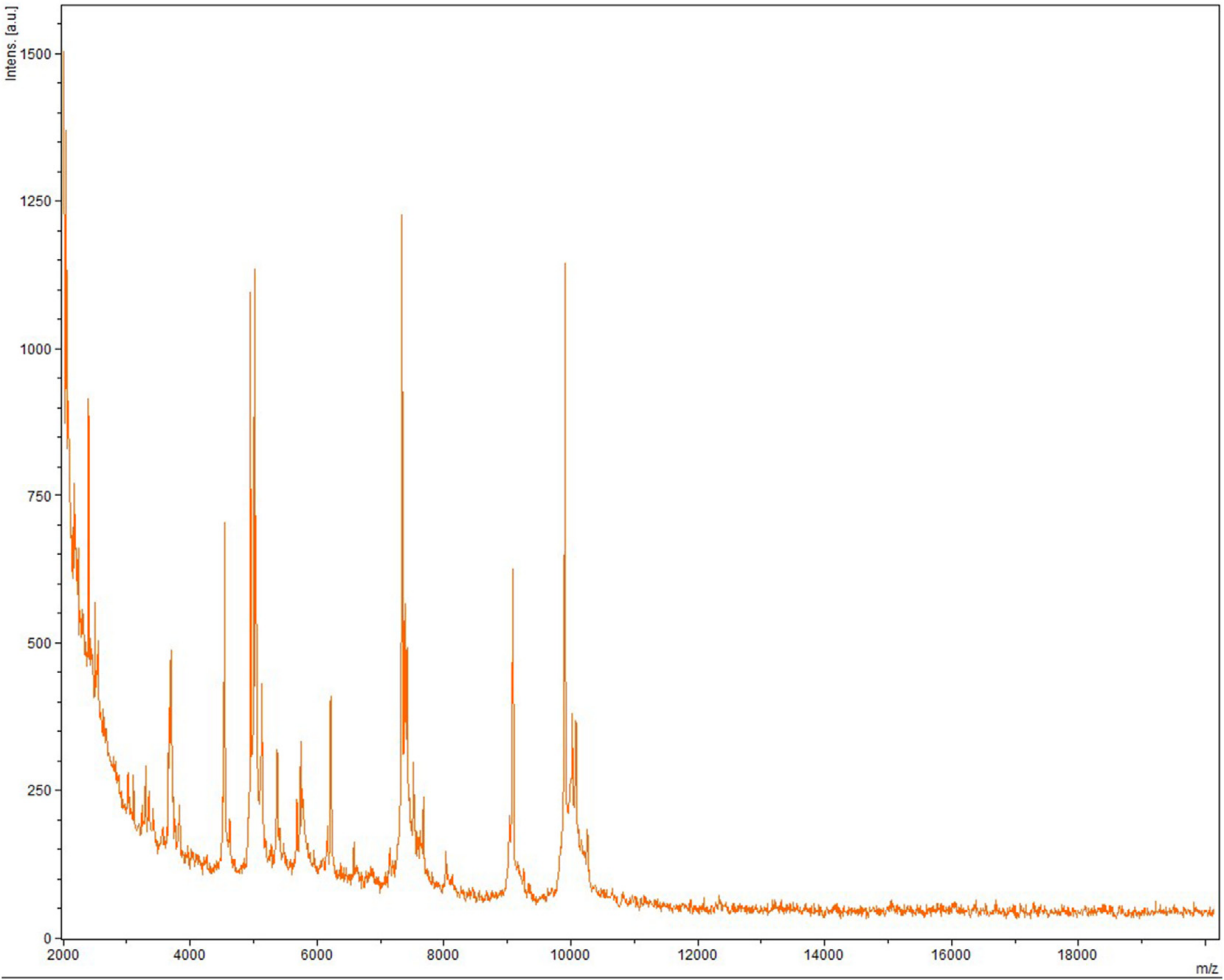
MALDI-TOF MS Reference mass spectrum. Spectra from 12 individual colonies were compared and a reference spectrum was generated.

**Fig. 2:**
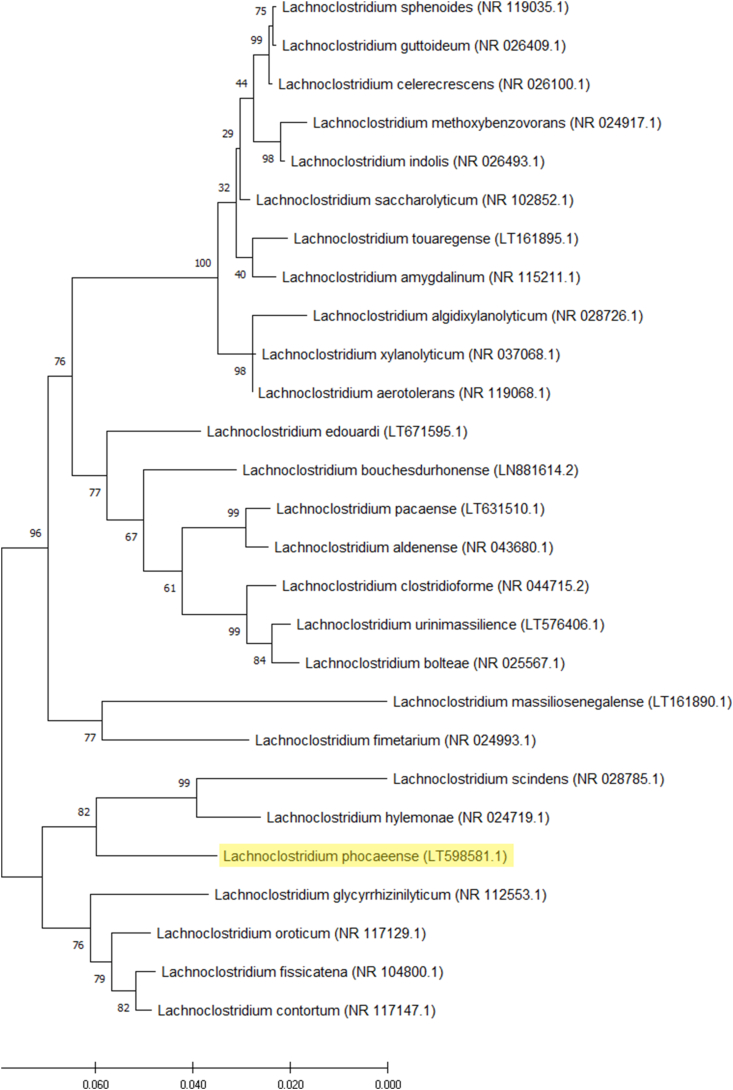
Phylogenetic tree analysis based on partial 16S ribosomal RNA sequences. Genbank accession numbers of partial 16S rRNA gene sequence are indicated in parenthesis. Sequences were aligned using CLUSTALW and the phylogenetic tree was obtained using the maximum likelihood bootstrap method and MEGA 7 software [20]. Numbers shown at the nodes are bootstrap percentages values obtained by 1000 times repetition analysis.

*Lachnoclostridium* species, named *phocaeense* strain Marseille-P3177 had a unique spectrum upon identification with MALDI-TOF-MS on a Microflex LT spectrometer (Bruker Daltonics, Bremen, Germany). The reference spectrum obtained (Fig. 1) was imported into our database (http://www.mediterranee-infection.com/article.php?larub=280&titre=urms-database). The *L*. *phocaeense* 16S rRNA gene exhibited 94.6% similarity with *Lachnoclostridium contortum* strain ATCC 25540 [11], a phylogenetically close species (Fig. 2). The 94.6% value is lower than the gene sequence threshold of 98.7% 16S rRNA recommended by Stackebrandt and Ebers [12] to characterize an isolated strain as a new bacterial species without DNA–DNA hybridization.

## Phenotypic and biochemical characterization

Strain Marseille-P3177 appears as translucent and whitish circular colonies with a diameter of 0.7–1 mm on a 5% sheep blood Columbia agar medium (BioMérieux, Marcy-l’Étoile, France). This species developed under anaerobic conditions at 37°C and for a period of 5 days of incubation [10].

**Fig. 3:**
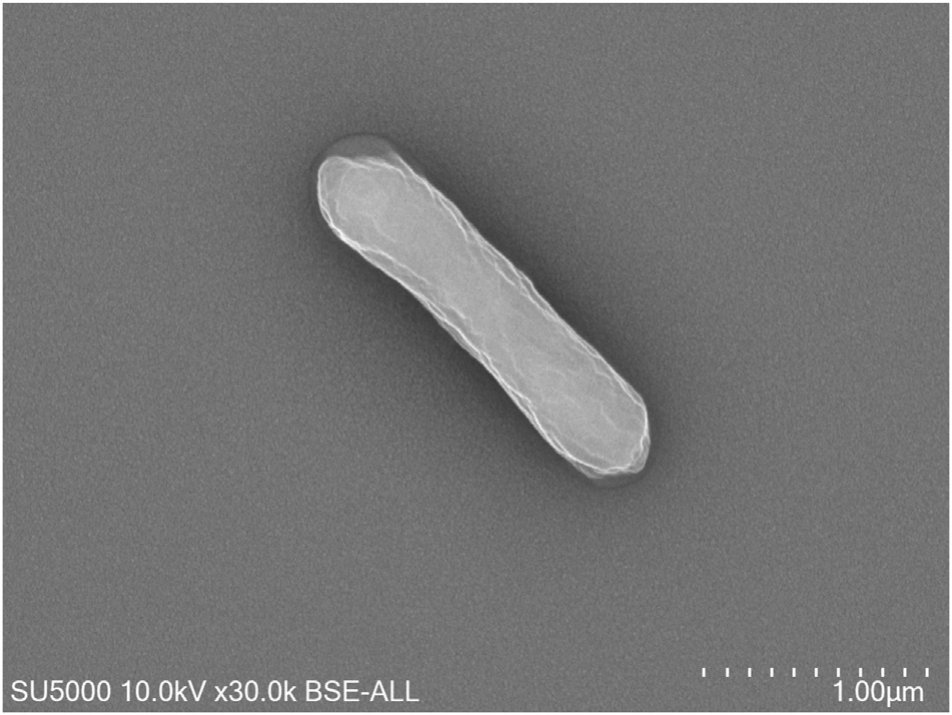
Transmission electron microscopy of *L. phocaeense* P3177 using GD6 using TechnaiG2 Cryo (FEI Company, Limeil-Brevannes, France).

Electron microscopy using GD6 and TechnaiG2 Cryo (FEI Company, Limeil-Brevannes, France) showed that *L*. *phocaeense* strain Marseille-P3177 is a Gram-positive bacillus (Fig. 3).

Biochemical characteristics of the isolated strain were determined using API ZYM and API 50CH (BioMérieux). Catalase assays (BioMérieux) and oxidase assays (Becton Dickinson, Le Pont de Claix, France) showed that this strain is oxidase and catalase negative. API ZYM revealed positive reactions for acid phosphatase, naphthol-AS-BI-phosphohydrolase, α-galactosidase, β-galactosidase, α-glucosidase and β-glucosidase. On the other hand, using API 50CH, acid production was observed in the presence of starch (Table 1).

**Table 1:** API 50CH and API ZYM biochemical tests of *Lachnoclostridium phocaeense* P3177

Test	Variable	Result
API 50 CH	Control	+
	Glycerol	—
	Erythrol	—
	d-Arabinose	—
	l-Arabinose	—
	d-Ribose	—
	d-Xylose	—
	l-Xylose	—
	d-Adonitol	—
	Methyl β-d-xylopyranoside	—
	d-Galactose	—
	d-Glucose	—
	d-Fructose	—
	d-Mannose	—
	l-Sorobose	—
	l-Rhamnose	—
	Dulcito	—
	Inositol	—
	d-Mannitol	—
	d-Sorbitol	—
	Methyl α-d-manopyranoside	—
	Methyl α-d-glucopyranoside	—
	*N*-Acetyl-glucosamine	—
	Amygladin	—
	Arbutin	—
	Esculin	—
	Salicin	—
	d-Cellobiose	—
	d-Maltose	—
	d-Lactose	—
	d-Melibiose	—
	d-Saccharose	—
	d-Trehalose	—
	Inulin	—
	d-Melezitose	—
	d-Raffinose	—
	Starch	+
	Glycogen	—
	Xylitol	—
	Gentiobiose	—
	d-Turanose	—
	d-Lyxose	—
	d-Tagatose	—
	d-Fucose	—
	l-Fucose	—
	d-Arabitol	—
	l-Arabitol	—
	Potassium gluconate	—
	Potassium 2-ketogluconate	—
	Potassium 5-ketogluconate	—
API ZYM	Alkaline phosphatase	—
	Esterase (C4)	—
	Esterase lipase (C8)	—
	Lipase (C14)	—
	Leucine arylamidase	—
	Valine arylamidase	—
	Cystine arylamidase	—
	Trypsin	—
	α-Chymotrypsin	—
	Acid Phosphatase	+
	Naphthalo-AS-BI-phosphohydrolase	+
	α-Galactosidase	+
	β-Galactosidase	+
	β-Glucuronidase	—
	α-Glucosidase	+
	β-Glucosidase	+
	*N*-Acetyl-glucosaminidase	—
	α-Mannosidase	—
	α-Fucosidase	—

Antibiotic susceptibility testing was done using E-test (BioMérieux) performed on Mueller–Hinton agar supplemented with 5% blood (BioMérieux). Interpretation of the results was done according to the European Committee on Antimicrobial susceptibility testing 2018 (EUCAST). The strain was susceptible to amoxicillin, cefotaxime, ertapenem, impipenem, meropenem, vancomycin, teicoplanin, metronidazole, trimethoprim-sulfamethoxazole, rifampicin and gentamicin; but resistant to ciprofloxacin, fosfomycin, colistin, doxycycline, oxofloxacin and erythromycin.

## Genome sequencing

Extracted genomic DNA of *L. phocaeense* P3177 was sequenced using MiSeq (Illumina, San Diego, CA, USA) with the mate-pair strategy. Assembly and annotation were performed with a pipeline of different softwares (Spades [13], Velvet [11], Soap Denovo [14], trimmed (Trimmomatic), MiSeq [15] software or untrimmed data (only MiSeq software) and XEGEN (http://www.xegen.fr/). To reduce assembly gaps, GapCloser was used. Scaffolds with depth value < 25% of the mean depth and <800 bp were removed. Using different criteria (number of N, number of scaffolds and N50), the best assembly was selected. Genome coverage was 125×. The predicted bacterial protein sequences for *L. phocaeense* in addition to the five complete genomes of *Lachnclostridium* available on NCBI were searched against the Clusters of Orthologous Groups (COG) database and NR database using blastp [16].

**Fig. 4:**
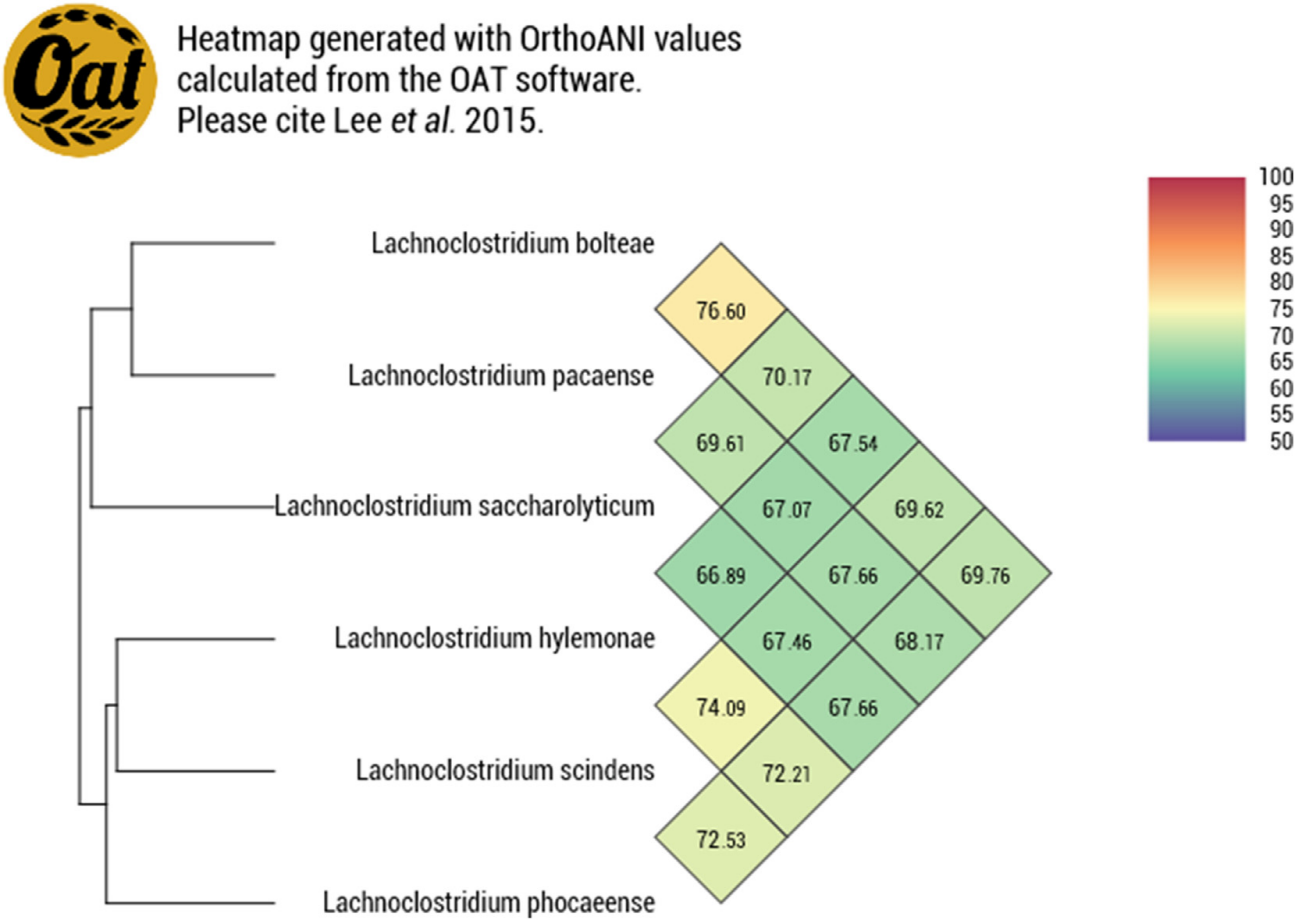
Heatmap generated with OrthoANI values calculated using the OAT software between *L. phocaeense* sp. nov. and other closely related species with standing in nomenclature.

The degree of genomic similarity of Marseille-P3177 with closely related species was estimated using the OrthoANI software [17]. Values among closely related species (Fig. 4) ranged from 67.07% between *Lachnoclostridium pacaense* and *Lachnoclostridium hylemonae* to 76.60% between *Lachnoclostridium bolteae* and *Lachnoclostridium pacaense*. When strain Marseille-P3177 was compared with these closely related species, values ranged from 67.66% with *L. saccharolyticum* to 72.53% with *L. scindens*.

## Genome description

**Fig. 5:**
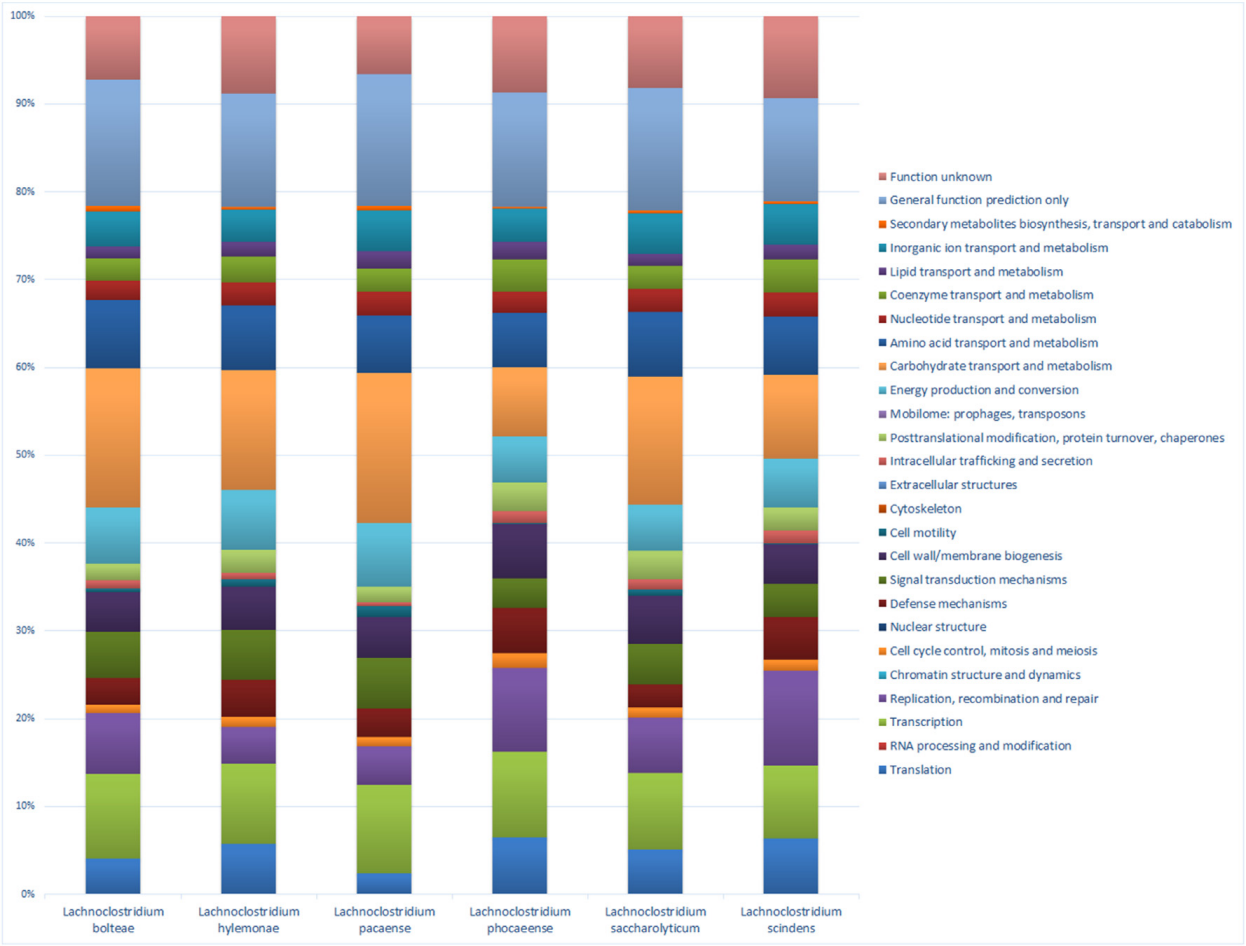
Functional distribution of COG categories in *L. phocaeense*, *L. hylemonae* (Genbank accession number NZ_CP036524.1), *L. pacaense* (Genbank accession number UOUF01000001.1), *L. saccharolyticum* (Genbank accession number NC_014376.1), *L. bolteae* (Genbank accession number NZ_CP022464.2) and *L. scindens* (Genbank accession number NZ_CP036170.1).

*Lachnoclostridium phocaeense* strain Marseille P3177 genome (GenBank accession number NZ_LT635479.1) is 3 500 754 bp long with 50.62% G + C content (Table 2). The genome coverage was 125×. Of the 3382 predicted genes, 3315 were protein-coding genes and 67 were RNAs (four genes were 5S rRNA, four genes were 16S rRNA, four genes were 23S rRNA, 55 genes were tRNA genes). A total of 2328 genes (70.23%) were assigned as putative function (by COGs or by NR blast). A total of 170 genes were identified as ORFans (5.13%). The remaining genes were annotated as hypothetical proteins (719 genes, 21.69%). Gene distribution into COG functional categories of *L. phocaeense* are presented in Table 3. The distribution of genes in COG categories was similar in all six species of *Lachnoclostridium* (Fig. 5).

**Table 2:** Genes and nucleotides content of the *Lachnoclostridium phoceense* genome

Variant	Number	% of the total
Size (bp)	3 500 754	100.0
G + C content (bp)	1 772 172	50.6
Total of genes	3382	100.0
RNA genes	67	2.0
Coding sequence size (bp)	3 152 738	90.1
Protein coding genes	3315	98.0
Protein associated to COGs	1905	57.5
Protein with peptide signal	300	9.0
Protein with transmembrane helices	733	22.1
Genes associated to mobilome	1259	38.0
Genes associated to virulence	531	16.0

Abbreviations: COGs, clusters of orthologous groups.

**Table 3:** Number of genes associated with the 25 general COG functional categories in *Lachnoclostridium phocaeense*

Code	Value	% of total	Description
[J]	195	5.882353	Translation
[A]	0	0	RNA processing and modification
[K]	201	6.0633483	Transcription
[L]	107	3.227753	Replication, recombination and repair
[B]	0	0	Chromatin structure and dynamics
[D]	40	1.2066365	Cell cycle control, mitosis and meiosis
[Y]	0	0	Nuclear structure
[V]	89	2.6847663	Defence mechanisms
[T]	101	3.0467572	Signal transduction mechanisms
[M]	101	3.0467572	Cell wall/membrane biogenesis
[N]	12	0.36199096	Cell motility
[Z]	0	0	Cytoskeleton
[W]	2	0.06033183	Extracellular structures
[U]	28	0.8446456	Intracellular trafficking and secretion
[O]	78	2.3529413	Post-translational modification, protein turnover, chaperones
[X]	48	1.4479638	Mobilome: prophages, transposons
[C]	111	3.3484166	Energy production and conversion
[G]	191	5.761689	Carbohydrate transport and metabolism
[E]	165	4.9773755	Amino acid transport and metabolism
[F]	72	2.1719458	Nucleotide transport and metabolism
[H]	115	3.4690802	Coenzyme transport and metabolism
[I]	63	1.9004526	Lipid transport and metabolism
[P]	78	2.3529413	Inorganic ion transport and metabolism
[Q]	24	0.7239819	Secondary metabolites biosynthesis, transport and catabolism
[R]	179	5.3996983	General function prediction only
[S]	98	2.9562595	Function unknown
—	1410	42.533936	Not in COGs

Abbreviations: COGs, clusters of orthologous groups.

Using the Bio-Edit interface, a BLAST search was conducted against ARG-ANNOT, a database for acquired antibiotic resistance genes (ARGs). The BLAST search was done under an e-value of 10^−5^, moderately stringent conditions for *in silico* ARG prediction [18]. ARG-ANNOT BLAST search revealed the presence of one resistance gene against tetracycline. This is in accordance with the antibiotic susceptibility testing performed, which showed that this strain was resistant to doxycycline. The bacteriocin database available in our research unit (Bacteriocins of the URMITE database BUR; available at http://drissifatima.wix.com/bacteriocins) was set up through the collection of all available sequences from NCBI and databases. Protein sequences from the aforementioned database allow the identification of bacteriocins from the human gut microbiota using BLASTp methodology [19]. Resistome analysis via this database showed the presence of 25 bacteriocin-associated genes.

## Description of *Lachnoclostridium phocaeense* sp. nov.

*Lachnoclostridium phocaeense* (pho.cae.en'se, L. neut. adj. phocaeense, referring to the town Phocaea, the Latin name of the city that was later named Marseille, in France, where the type strain was first isolated). *Lachnoclostridium phocaeense* strain Marseille-P3177 is a new species in the genus *Lachnoclostridium* that was isolated from a 51-year-old woman's urine sample after kidney transplantation in Marseille. The species' optimal growth conditions are 37°C for 5 days under anaerobic conditions. Colonies are 0.7–1 mm in diameter on blood-supplemented agar. *Lachnoclostridium phocaeense* is a strictly anaerobic Gram-positive rod. It is also catalase and oxidase negative.

## Funding

This work was supported by the French Government under the *Investissements d’avenir* (Investments for the Future) programme managed by the Agence Nationale de la Recherche (ANR, fr: National Agency for Research), (reference: Méditerranée Infection 10-IAHU-03).

## Conflicts of interest

There are no conflicts of interest or financial disclosures for any authors.

## References

[1] Yutin N., Galperin M.Y. (2013). A genomic update on clostridial phylogeny: Gram-negative spore formers and other misplaced *clostridia*. Environ Microbiol.

[2] Lopetuso L.R., Scaldaferri F., Petito V., Gasbarrini A. (2013). Commensal *Clostridia*: leading players in the maintenance of gut homeostasis. Gut Pathog.

[3] Guo P., Zhang K., Ma X., He P. (2020). *Clostridium* species as probiotics: potentials and challenges. J Anim Sci Biotechnol.

[4] Fraher M.H., O'Toole P.W., Quigley E.M. (2012). Techniques used to characterize the gut microbiota: a guide for the clinician. Nat Rev Gastroenterol Hepatol.

[5] Lagier J.C., Edouard S., Pagnier I., Mediannikov O., Drancourt M., Raoult D. (2015). Current and past strategies for bacterial culture in clinical microbiology. Clin Microbiol Rev.

[6] Turnbaugh P.J., Ley R.E., Hamady M., Fraser-Liggett C.M., Knight R., Gordon J.I. (2007). The human microbiome project. Nature.

[7] Lagier J.C., Khelaifia S., Alou M.T., Ndongo S., Dione N., Hugon P. (2016). Culture of previously uncultured members of the human gut microbiota by culturomics. Nat Microbiol.

[8] Ramasamy D., Mishra A.K., Lagier J.C., Padhmanabhan R., Rossi M., Sentausa E. (2014). A polyphasic strategy incorporating genomic data for the taxonomic description of novel bacterial species. Int J Syst Evol Microbiol.

[9] Fournier P.E., Lagier J.C., Dubourg G., Raoult D. (2015). From culturomics to taxonomogenomics: a need to change the taxonomy of prokaryotes in clinical microbiology. Anaerobe.

[10] Brahimi S., Cadoret F., Fournier P.E., Moal V., Raoult D. (2017). *Lachnoclostridium urinimassiliense*' sp. nov. and '*Lachnoclostridium phocaeense*' sp. nov., two new bacterial species isolated from human urine after kidney transplantation. New Microbe. New Infect.

[11] Zerbino D.R., Birney E. (2008). Velvet: algorithms for de novo short read assembly using de Bruijn graphs. Genome Res.

[12] Stackebrandt E., Ebers J. (2006). Taxonomic parameters revisited: tarnished gold standards. Microbiol Today.

[13] Bankevich A., Nurk S., Antipov D., Gurevich A.A., Dvorkin M., Kulikov A.S. (2012). SPAdes: a new genome assembly algorithm and its applications to single-cell sequencing. J Comput Biol.

[14] Luo R., Liu B., Xie Y., Li Z., Huang W., Yuan J. (2012). SOAPdenovo2: an empirically improved memory-efficient short-read de novo assembler. Gigascience.

[15] Bolger A.M., Lohse M., Usadel B. (2014). Trimmomatic: a flexible trimmer for Illumina sequence data. Bioinformatics.

[16] Anani H., Abou Abdallah R., Chelkha N., Fontanini A., Ricaboni D., Mailhe M. (2019). Draft genome and description of *Merdibacter massiliensis* gen.nov., sp. nov., a new bacterium genus isolated from the human ileum. Sci Rep.

[17] Lee I., Ouk Kim Y., Park S.C., Chun J. (2016). OrthoANI: an improved algorithm and software for calculating average nucleotide identity. Int J Syst Evol Microbiol.

[18] Gupta S.K., Padmanabhan B.R., Diene S.M., Lopez-Rojas R., Kempf M., Landraud L. (2014). ARG-ANNOT, a new bioinformatic tool to discover antibiotic resistance genes in bacterial genomes. Antimicrob Agents Chemother.

[19] Drissi F., Buffet S., Raoult D., Merhej V. (2015). Common occurrence of antibacterial agents in human intestinal microbiota. Front Microbiol.

[20] Kumar S., Stecher G., Tamura K. (2016). MEGA7: molecular evolutionary genetics analysis version 7.0 for bigger datasets. Mol Biol Evol.

